# Supplementary material for: Modeling glioblastoma heterogeneity as a dynamic network of cell states
Source: Mol Syst Biol. 2021 Sep 16;17(9):e10105. doi: 10.15252/msb.202010105 (PMC8444284; doi:10.15252/msb.202010105)
Supplement: Supplementary file 6 — Source Data for Figure 5 [file MSB-17-e10105-s004.zip › Figure5A_sourcedata/GSEA_3071/hallmarks_stateI.GseaPreranked.1623742531165/HALLMARK_HEDGEHOG_SIGNALING.html]

Details for gene set HALLMARK\_HEDGEHOG\_SIGNALING[GSEA]

|  || Dataset | state13071 |
| Phenotype | NoPhenotypeAvailable |
| Upregulated in class | na\_neg |
| GeneSet | HALLMARK\_HEDGEHOG\_SIGNALING |
| Enrichment Score (ES) | -0.26818871 |
| Normalized Enrichment Score (NES) | -0.9992757 |
| Nominal p-value | 0.44867548 |
| FDR q-value | 0.6756538 |
| FWER p-Value | 1.0 |
Table: GSEA Results Summary

  

Fig 1: Enrichment plot: HALLMARK\_HEDGEHOG\_SIGNALING      
 Profile of the Running ES Score & Positions of GeneSet Members on the Rank Ordered List

  

| PROBE | GENE SYMBOL | GENE\_TITLE | RANK IN GENE LIST | RANK METRIC SCORE | RUNNING ES | CORE ENRICHMENT || 1 | PTCH1 |  |  | 7 | 1.951 | 0.1003 | Yes |
| 2 | NRP1 |  |  | 81 | 1.180 | 0.1413 | Yes |
| 3 | THY1 |  |  | 94 | 1.114 | 0.1964 | Yes |
| 4 | TLE3 |  |  | 126 | 0.968 | 0.2382 | Yes |
| 5 | L1CAM |  |  | 369 | 0.617 | 0.2012 | No |
| 6 | GLI1 |  |  | 452 | 0.547 | 0.2064 | No |
| 7 | HEY1 |  |  | 497 | 0.517 | 0.2209 | No |
| 8 | ETS2 |  |  | 611 | 0.446 | 0.2119 | No |
| 9 | NF1 |  |  | 617 | 0.445 | 0.2338 | No |
| 10 | MYH9 |  |  | 701 | 0.411 | 0.2315 | No |
| 11 | RASA1 |  |  | 935 | 0.342 | 0.1827 | No |
| 12 | UNC5C |  |  | 1024 | 0.323 | 0.1744 | No |
| 13 | PML |  |  | 1211 | 0.286 | 0.1361 | No |
| 14 | VEGFA |  |  | 1644 | -0.273 | 0.0265 | No |
| 15 | CDK5R1 |  |  | 1879 | -0.302 | -0.0248 | No |
| 16 | HEY2 |  |  | 1882 | -0.302 | -0.0095 | No |
| 17 | CRMP1 |  |  | 2324 | -0.377 | -0.1162 | No |
| 18 | TLE1 |  |  | 2502 | -0.415 | -0.1452 | No |
| 19 | CNTFR |  |  | 2697 | -0.471 | -0.1761 | No |
| 20 | RTN1 |  |  | 2775 | -0.496 | -0.1722 | No |
| 21 | DPYSL2 |  |  | 3104 | -0.680 | -0.2305 | No |
| 22 | SCG2 |  |  | 3130 | -0.703 | -0.2008 | No |
| 23 | CDK6 |  |  | 3366 | -1.085 | -0.2113 | No |
| 24 | NRCAM |  |  | 3495 | -2.259 | -0.1294 | No |
| 25 | NRP2 |  |  | 3502 | -2.549 | 0.0026 | No |
Table: GSEA details [plain text format]

  

Fig 2: HALLMARK\_HEDGEHOG\_SIGNALING: Random ES distribution      
 Gene set null distribution of ES for **HALLMARK\_HEDGEHOG\_SIGNALING**
